# Prevalence of Herpes B Virus in Wild Long-Tailed Macaques, Thailand, 2018–2024

**DOI:** 10.3201/eid3104.241197

**Published:** 2025-04

**Authors:** Krittiga Sapkanarak, Jiro Yasuda, Murasaki Amano, Suthirote Meesawat, Taratorn Kemthong, Titiporn Kaikaew, Suchinda Malaivijitnond

**Affiliations:** Chulalongkorn University Faculty of Science, Bangkok, Thailand (K. Sapkanarak, T. Kaikaew, S. Malaivijitnond); Nagasaki University, Nagasaki, Japan (J. Yasuda, M. Amano); National Primate Research Center of Thailand, Saraburi, Thailand (S. Meesawat, T. Kemthong, S. Malaivijitnond)

**Keywords:** herpes B virus, Burmese long-tailed macaque, common long-tailed macaque, cynomolgus macaque, seropositive, shedding, viruses, zoonoses, Thailand

## Abstract

Herpes B virus (B virus) is an enigmatic zoonotic virus that has caused severe neurologic symptoms in humans exposed to captive macaques used for experimentation. We examined 864 wild long-tailed macaques from 22 locations across Thailand for B virus infection. All 22 macaque populations tested positive for B virus antibodies; seropositivity ranged from 25% to 100%. B virus shedding was detected in 9 (1.04%) oral swab samples by using quantitative PCR of the virus UL29 gene. We phylogenetically analyzed partial genome sequences of B virus (US5-US6 genes) from 6 of the PCR-positive samples. All 6 sequences were clustered in clade II, which includes B virus strains from rhesus, Japanese, and long-tailed macaques, suggesting co-evolution of B virus with macaques. Continued surveillance and sequencing of B virus in macaque populations will be needed to prevent B virus transmission to humans and to develop appropriate vaccines to prevent human B virus infections.

Herpes B virus (B virus; also known as *Macacine alphaherpesvirus* 1 or *Simplexvirus macacinealpha* 1) is a double-stranded DNA virus that predominantly infects macaques in Asia, particularly long-tailed (*Macaca fascicularis*) and rhesus (*M. mulatta*) macaques (*1*,*2*). The B virus envelope contains the glycoproteins gB, gC, gD, gE, gG, gH, gL, and gM (*3*) that promote binding to and entry into host cells; virus DNA is released into the host nucleus and virus replication commences. Virus shedding occurs as B virus is transported to the host cell surface, where it forms vesicles primarily observed in oral and genital mucosae of infected animals. B virus transmission between animals occurs through bites, scratches, saliva, or urine. B virus establishes lifelong latency in the trigeminal and lumbosacral sensory ganglia, characterized by limited virus gene expression and no replication (*4*). Reactivation is triggered by several factors, such as environmental changes, stress, immune suppression, social challenges, and migration (*5*).

After B virus infection, virus glycoproteins can induce host humoral immune responses and antibody development. Virus-specific IgM typically emerges 7–10 days postinfection, followed by IgG at 14–21 days, peaking at 30–40 days (*6*). In long-tailed and rhesus macaques, the highest incidences of infection are commonly attributed to sexual activity among adults during the breeding season (*7**–**9*). Infected macaques typically exhibit asymptomatic or mild symptoms, and the virus remains latent within the sensory ganglia. Although extremely rare, B virus infection in humans can lead to severe and potentially fatal neurologic complications (*10*). When the virus spreads to the brain, encephalomyelitis, respiratory failure, and coma are more frequently experienced in humans; 70%–80% of humans who contract B virus have a high probability of death. However, the mortality rate can be decreased by antiviral drug therapy (*11*). Although the disease was first identified in 1932, only 50 human cases have been reported, and 21 patients have died. 

B virus infections in humans are generally caused by contact with captive or laboratory macaques. For example, a veterinary surgeon in China who conducted necropsies on 2 deceased laboratory monkeys in 2021 exhibited symptoms consistent with B virus infection, tested positive for B virus, and died 1 month later (*12*).

Several methods for B virus detection have been developed. Although virus isolation is the standard detection method, the process requires a Biosafety Level 3 or 4 facility to culture B virus (*13*). ELISA is widely used for serologic detection of B virus antibodies; however, it does not measure virus shedding. To detect virus shedding, quantitative PCR (qPCR) and loop-mediated isothermal amplification (LAMP) methods are effective for identifying B virus DNA (*14*).

In Thailand, human–*M. fascicularis* macaque interactions leading to bites, scratches, and mucosal splashes regularly occur throughout the country, and yet no cases of B virus infections among exposed humans have been reported (*15*–*17*). *M. fascicularis* has been classified into 2 subspecies in Thailand: Burmese (*M. fascicularis* subspecies *aurea*) and common (*M. fascicularis* subsp. *fascicularis*) long-tailed macaques (*18*). Although they belong to the same species, their genetics are distinguishable (*18*–*20*). In 2022, a total of 649 free-ranging *M. fascicularis* macaques from 26 urban areas in Thailand were captured and tested for B virus infection by using real-time PCR with primers designed to detect the glycoprotein G gene. However, no positive cases were detected; nonshedding B virus infections in those monkeys was the suspected cause of detection failure (*21*). We recently developed a qPCR method to detect B virus (*14*), and we suggest that the real-time PCR used in the previous study (*21*) might not accurately detect B virus or reflect B virus infection and shedding in wild macaques. We investigated B virus seroprevalence and shedding in wild *M. fascicularis* macaques throughout Thailand and analyzed associations with macaque age, sex, and subspecies status. We captured macaques from 22 populations across Thailand and tested for B virus antibodies and sequenced isolated B virus genomes.

## Methods

### Ethics Statement

The National Primate Research Center of Thailand-Chulalongkorn University (NPRCT-CU) Animal Care and Use Committee approved all animal procedures (protocol nos. 2075007 and 2475006). The Department of National Parks, Wildlife and Plant Conservation, Thailand, approved monkey capture and specimen collections.

### Study Sites and Sample Collections

We investigated B virus seroprevalence and shedding in 864 macaques from 18 *M. fascicularis* subsp. *fascicularis* and 4 *M. fascicularis* subsp. *aurea* populations that lived freely in human–macaque interface areas (Table 1). We identified the subspecies according to morphologic characteristics and distribution range (*18*,*20*,*22*,*23*). We captured animals during September 2018–May 2024 by using iron mesh traps or automatic box traps and then anesthetized them by intramuscular injection of 2–5 mg/kg body weight of Zoletil mixed with 20–50 μg/kg body weight of dexmedetomidine (*24*). We identified and recorded sex and estimated age on the basis of dental eruption patterns (*25*). We used 3 age classes: adult, >6 years; subadult, 3–6 years; and juvenile, 1–3 years. We collected blood from the femoral vein and mixed it with heparin (LEO Pharma, https://www.leo-pharma.com). We centrifuged the blood at 1,000 × *g* for 15 minutes, collected plasma, and stored the sample at −20°C until use for detecting B virus antibodies by ELISA (commercially available for *M. fascicularis* but slightly modified for this study). We collected oral swab samples by using nylon flock swabs (Hangzhou Rollmed Co., Ltd., https://www.rollmed.net) and preserved those in 1.0 mL DNA/RNA Shield (Zymo Research, https://www.zymoresearch.com) until use for B virus genome detection by LAMP and qPCR (primers amplifying conserved sequences among all B virus strains were designed and validated) (*14*). After sample collection, we injected the monkeys intramuscularly with atipamezole hydrochloride (Zoetis, https://www.zoetis.com), kept them in cages, and released them back into their native habitats after full recovery.

**Table 1:** Location, code, geographic coordinates, and habitat type of 22 populations of macaques in study of prevalence of herpes B virus in Thailand, 2018–2024*

Macaque location	Code	GPS coordinates	Habitat type
Common long-tailed macaque, *Macaca fascicularis* subspecies *fascicularis*			
Wat Haad Moon	WHM	16°30′N, 100°16′E	Mainland
Kao Nor	KN	15°57′N, 99°52′E	Mainland
Wat Tham Thep Ban Dan	WTT	15°44′N, 101°02′E	Mainland
Muang Ling Ban Wan	MBW	15°37′N, 104°18′E	Mainland
Wat Mueang Khaen Yai	WMK	15°36′N, 104°21′E	Mainland
Wat Phikun Ngam	PKN	15°27′N, 100°05′E	Mainland
Wat KuPhra Ko Na	WKN	15°21′N, 104°13′E	Mainland
Wat Kai	WK	14°50′N, 100°52′E	Mainland
Khao Laem Pu Chao	LPC	13°39′N, 100°52′E	Mainland
Wat Tham Khao Chakan	WKCK	13°39′N, 102°05′E	Mainland
Kao Ngu	KNG	13°34′N, 99°46′E	Mainland
Bang Ta Boon	BTB	13°15′N, 99°56′E	Mainland
Wat Tham Khao Cha Ang On	CAO	13°12′N, 101°39′E	Mainland
Wat Khao Wong Khot	KWK	12°52′N, 101°49′E	Mainland
Koh Ped	KPE	12°45′N, 100°50′E	Island
Wat Suwan Kuha	WSK	8°25′N, 98°28′E	Mainland
Wat Khao Keaw wichian	WKK	8°12′N, 100°05′E	Mainland
Khao Chaison	KCS	7°27′N, 100°07′E	Mainland
Burmese long-tailed macaque, *M. fascicularis* subsp. *aurea*			
Tham Pra Khayang	TPK	10°19′N, 98°45′E	Mangrove forest
World War Museum	WWM	10°10′N, 98°43′E	Mangrove forest
Mangrove Forest Research Center	MFRC	9°87′N, 98°60′E	Mangrove forest
Moo Koh Ranong	MKR	9°52′N, 98°35′E	Mangrove forest

*GPS, global positioning system.

### Detection of B Virus Antibodies by ELISA

We tested all plasma samples for B virus IgG by using a simian herpes virus ELISA kit (VRL Diagnostics, https://www.vrlsat.com). In brief, we coated B virus antigen in microplate wells. We diluted plasma samples (100 μL) to 1:50 with diluent containing 5% nonfat dry milk, added the diluted sample to the microplate, and incubated at 37°C for 30 minutes. After washing 5 times with 300 μL of phosphate-buffered saline with Tween-20, we added 100 μL of 1:100 dilution of horseradish peroxidase–labeled anti-IgG, incubated at 37°C for 30 minutes, and then washed 5 times with 300 μL of buffer. To visualize enzyme activity, we added 100 μL of horseradish peroxidase substrate per well and incubated the microplate for 30 minutes at room temperature, then added 25 μL of stop solution. We measured and recorded the optical density (OD) at 405 nm. 

The cutoff value for positive ELISA results was OD >0.300 according to the manufacturer. However, we did not use that cutoff value for *M. fascicularis* macaques because the value was too low, and nearly all tested animals showed a positive result. We established a new ELISA cutoff value and standard curve. From the NPRCT-CU plasma bank, we selected 20 B virus–negative and 40 B virus–positive plasma samples collected from *M. fascicularis* macaques; the samples were previously submitted for B virus ELISA testing at the Corporation for Production and Research of Laboratory Primates (https://www.primate.or.jp), Japan, during 2017–2018. We pooled the 20 B virus–negative samples or the 40 B virus–positive samples to establish the standard curve. We serially diluted the pooled positive sample 4-fold from 1:16 to 1:65,536 to cover the entire OD range (0.082–3.682) observed for B virus-positive plasma samples from *M. fascicularis* macaques in the NPRCT-CU plasma bank. We designated the OD value from the 1:16 dilution as 100 arbitrary units (AU) of B virus IgG and prepared a standard curve by using GraphPad version 10.0.3 (https://www.graphpad.com) (Appendix Figure, panel A). To determine interassay variation, we calculated the percentage coefficient of variation (%CV) for low (%CV 5.2%; OD +SD 1.82 +0.09), medium (%CV 4.9%; OD 3.19 +0.16), and high (%CV 5.3%; OD 3.56 +0.19) concentrations of quality control plasma samples; we performed those assays 9 times for each concentration. We determined the new cutoff OD was >0.765 for *M. fascicularis* macaques.

### Detection of B Virus Shedding by LAMP and qPCR

We extracted DNA from oral swab samples by using a QIAamp DNA Mini Kit (QIAGEN, https://www.qiagen.com) and detected the B virus genome by using LAMP and qPCR as described previously (*14*). We selected specific primers to amplify conserved regions of the B virus UL27 (for LAMP) and UL29 (for qPCR) genes. For positive controls, we used 248-bp sequences of UL27 (position 1850–2097) for LAMP and 257-bp sequences of UL29 (position 1093–1349) for qPCR. We created a qPCR standard curve by using 10-fold serial dilutions of the UL29 positive control (Appendix Figure, panel B) and calculated B virus DNA copy numbers for each sample; we set the cycle threshold cutoff at 40.

To confirm positive B virus qPCR results for oral swab samples, we performed nested PCR by using 2 primer sets specific for conserved regions of UL29 (562 bp) and UL27 (486 bp) (Table 2). The second (nested) primer set amplified a sequence within the first amplicon, but the PCR conditions were identical. The 25 μL reactions consisted of 5 μL of 5X PrimeSTAR GXL Buffer, 2 μL of dNTP mixture, 1 μL of 10× primer mixture, 0.5 μL of DNA polymerase (Takara Bio, https://www.takarabio.com), 15.5 μL of nuclease-free water (Invitrogen/Thermo Fisher Scientific, https://www.thermofisher.com), and 1 μL of extracted DNA. We conducted PCR by using a thermal cycle program of 10 seconds at 98°C, 15 seconds at 55°C, and then 30 cycles of 35 seconds at 68°C. Before sequencing, we purified amplicons by using the QIAquick Gel Extraction Kit (QIAGEN) after electrophoresis on a 1% agarose gel and visualization by ultraviolet transillumination. We analyzed sequences of B virus strains by using BLAST (https://blast.ncbi.nlm.nih.gov) and used the complete genome sequence of B virus isolate E90-136 obtained from GenBank (accession no. KJ566591.2) as a reference. We assembled and analyzed sequencing data by using MEGA 7 (https://www.megasoftware.net) and BioEdit version 7.2.5 (https://thalljiscience.github.io).

**Table 2:** PCR primers specific for B virus sequences in study of prevalence of herpes B virus in wild long-tailed macaques, Thailand, 2018–2024*

Primer set	Primer names	Primer sequences, 5′ → 3′
UL27, first PCR	UL27_F1	ACGTCATCATGCAGAACTCG
	UL27_R1	GGTTGGAGAGGAAGGAGGAC
UL27, second PCR	UL27_F2	ACGTCATCATGCAGAACTCG
	UL27_R2	GGCCCTCGAAGAAGGAGTAG
UL29, first PCR	UL29_F1	CAGTAGCGCAGGATCTGGTTG
	UL29_R1	GCGTGATGGACCTCTTYAACA
UL29, second PCR	UL29_F2	CAGGATCTGGTTGGCCATGTAG
	UL29_R2	GCGTGATGGACCTCTTYAACA
US5-US6, first PCR	US5-US6_F1	CGTTTCCTCCCGTGGACTTC
	US5-US6_R1	GTCTGGAACGGGTTCTCCAC
US5-US6, second PCR	US5-US6_F2	CTGACCCTGGCCGCCATG
	US5-US6_R2	GGTCCGTCTTCTGCTCCAGCG

*Primers were designed to detect conserved regions within the UL27 and UL29 genes and confirm the positive cases identified by using quantitative PCR and loop-mediated isothermal amplification and to detect hypervariable regions (US5-US6 genes) for phylogenetic analysis.

### Phylogenetic Analysis of Partial B Virus Sequences

After qPCR analysis, we performed another nested PCR on B virus–positive oral swab samples by using primers specific for hypervariable regions of US5 (encodes gJ) through US6 (encodes gD) genes (Table 2). PCR conditions were similar to those described previously, except the extension phase was 44 seconds at 68°C. We purified the PCR products by using the QIAquick Gel Extraction Kit and submitted them for sequencing. We assembled and analyzed the sequences as previously described. For phylogenetic analysis, we used B virus sequences isolated from *M. mulatta* (rhesus macaque) (GenBank accession nos. AF083210.1, AF082808.1, AF082804.1, AF082806.1, and AF082812.1), *M. fuscata* (Japanese macaque) (accession nos. AF082814.1 and AF082813.1), *M. fascicularis* (accession no. AF082811.1), *M. silenus* (lion-tailed macaque) (accession no. AF241218.1), and *M. nemestrina* (southern pig-tailed macaque) (accession nos. AF082807.1 and AF082805.1) as references. 

We conducted phylogenetic analysis of B virus sequences by using both Bayesian and maximum-likelihood methods. For Bayesian analysis, we selected the Hasegawa-Kishino-Yano plus proportion of invariable sites model according to the Bayesian information criterion by using MEGA 5.2. We performed the Bayesian analysis by using the Markov chain Monte Carlo algorithm via MrBayes 3.1.7 (https://nbisweden.github.io/MrBayes) and conducted 2 independent runs with >10 million generations per run, collecting parameters every 1,000 generations. We confirmed convergence when the average SD of split frequencies was <0.01 and the potential scale reduction factors for all parameters were ≈1.0. We discarded the first 25% of data (2,500 trees) from each run as burn-in and combined the remaining data to produce a 50% majority rule consensus tree with posterior probabilities on each branch. We conducted maximum-likelihood analysis by using MEGA 7 with 1,000 bootstrap replicates and visualized the results by using FigTree version 1.4.4 (http://tree.bio.ac.uk).

### Statistical Analyses

We determined prevalence rates and 95% CIs for differences in prevalence and shedding according to sex, age class, and subspecies level of *M. fascicularis* by using a generalized linear model. We conducted statistical analyses by using RStudio version 2024.04.0+735 (https://www.rstudio.com). We considered a p value of <0.05 as statistically significant.

## Results

### Seroprevalence 

The seropositive rate for B virus IgG in *M. fascicularis* macaques in Thailand was 78.5% (678/864) (Table 3). All 22 macaque populations had B virus–seropositive cases, ranging in frequency from 25% to 100%. The macaque population in Wat Tham Thep Ban Dan (WTT) had 100% B virus seropositive cases and the highest B virus antibody concentration of 91.65 AU. The population in Muang Ling Ban Wan had 25% seropositive cases; the antibody concentration was the lowest (0.071–4.995 AU). When analyzed separately according to subspecies, the seroprevalence was comparable between *M. fascicularis* subsp. *fascicularis* (78.9% [609/772]) and *M. fascicularis* subsp. *aurea* (75.0% [69/92]) macaques.

**Table 3:** Number of animals with B virus infection detected by using ELISA, qPCR, and LAMP in study of prevalence of herpes B virus in wild long-tailed macaques, Thailand, 2018–2024*

Macaque location	Code	No. animals	ELISA, no. (%)	Range of antibody concentration, AU	qPCR, no. (%)	LAMP, no. (%)
Common long-tailed macaque, *Macaca fascicularis* subspecies *fascicularis*						
Wat Haad Moon	WHM	27	24 (88.9)	0.07–21.91	0 (0)	0 (0)
Kao Nor	KN	75	71 (94.7)	0.05–42.34	0 (0)	0 (0)
Wat Tham Thep Ban Dan	WTT	55	55 (100.0)	1.99–91.65	1 (1.81)	0 (0)
Muang Ling Ban Wan	MBW	4	1 (25.0)	0.07–5.00	0 (0)	0 (0)
Wat Mueang Khaen Yai	WMK	60	52 (86.7)	0.03–13.43	0 (0)	0 (0)
Wat Phikun Ngam	PKN	9	8 (88.9)	0.07–27.03	0 (0)	0 (0)
Wat KuPhra Ko Na	WKN	77	59 (76.6)	0.01–35.83	3 (3.89)	3 (3.89)
Wat Kai	WK	55	42 (76.4)	0.03–45.49	0 (0)	0 (0)
Khao Laem Pu Chao	LPC	10	7 (70.0)	0.04–13.02	0 (0)	0 (0)
Wat Tham Khao Chakan	WKCK	50	42 (84.0)	0.03–50.94	0 (0)	0 (0)
Kao Ngu	KNG	70	63 (90.1)	0.02–40.56	0 (0)	0 (0)
Bang Ta Boon	BTB	45	28 (62.2)	0.02–11.49	1 (2.22)	1 (2.22)
Wat Tham Khao Cha Ang On	CAO	42	27 (64.3)	0.05–18.85	2 (4.76)	2 (4.76)
Wat Khao Wong Khot	KWK	47	29 (61.7)	0.03–36.25	0 (0)	0 (0)
Koh Ped	KPE	35	31 (88.6)	0.12–11.15	0 (0)	0 (0)
Wat Suwan Kuha	WSK	28	26 (92.9)	0.05–78.11	0 (0)	0 (0)
Wat Khao Keaw wichian	WKK	29	18 (62.1)	0.05–46.74	0 (0)	0 (0)
Khao Chaison	KCS	54	26 (48.1)	0.03–35.34	0 (0)	0 (0)
Total	NA	772	609 (78.9)	0.01–91.65	7 (0.90)	6 (0.77)
Burmese long-tailed macaque, *M. fascicularis* subsp. *aurea*						
Tham Pra Khayang	TPK	27	23 (85.1)	0.0529.930	1 (3.70)	1 (3.70)
World War Museum	WWM	31	17 (54.8)	0.02–19.31	0 (0)	0 (0)
Mangrove Forest Research Center	MFRC	23	20 (86.9)	0.04–17.73	1 (4.35)	1 (4.35)
Moo Koh Ranong	MKR	11	9 (81.8)	0.08–13.33	0 (0)	0 (0)
Total	NA	92	69 (75.0)	0.02–29.93	2 (2.17)	2 (2.17)
Grand total	NA	864	678 (78.5)	0.01–91.65	9 (1.04)	8 (0.92)

*AU, arbitrary unit; LAMP, loop-mediated isothermal amplification; NA, not applicable; qPCR, quantitative PCR.

Antibody seroprevalence was not significantly different between male (77.5% [409/528]) and female (80.1% [269/336]) macaques (Table 4). In contrast, the seropositive rate was significantly dependent on the age class (p<0.001). Adult animals had higher B virus prevalence at 95.9% (377/393), followed by subadults at 84.5% (240/284) and juveniles at 32.6% (61/187).

**Table 4:** Number of wild long-tailed macaques seropositive for B virus classified by age and sex in study of prevalence of herpes B virus in Thailand, 2018–2024*

Age class	No. positive/total (%, 95% CI)		
	Male	Female	Total
Adult	228/240 (95.5, 83.3–101.5)	149/153 (97.4, 70.4–99.9)	377/393 (95.9, 80.4–97.2)
Subadult	144/177 (81.4, 60.6–86.6)	96/107 (89.7, 54.7–89.4)	240/284 (84.5, 62.4–83.3)
Juvenile	37/111(36.6, 28.8–78.6)	24/76 (31.6, 6.7–34.4)	61/187 (32.6, 14.5–34.7)
Total	409/528 (77.5, 55.1–86.1)	269/336 (80.1, 59.8–92.6)	678/864 (78.5, 76.1–86.9)

*B virus was detected by using ELISA. Significant differences in seropositivity were observed among 3 age classes (p<0.05).

### Detection of B Virus Shedding

The B virus shedding detection rates were 1.04% (9/864 animals) by qPCR and 0.92% (8/864) by LAMP (Table 3). qPCR had a slightly higher sensitivity for B virus DNA detection. In 1 juvenile monkey (no. WTT47), qPCR detected 9 virus DNA copies, whereas LAMP had a negative result (Table 5). In 1 subadult monkey (no. CAO38) with only 7 virus DNA copies detected by qPCR, LAMP showed a positive result but had the longest amplification time of 24 minutes, 46 seconds. However, when virus loads were high, LAMP only required 13–16 minutes for reaction completion (Table 5).

**Table 5:** Detection of B virus DNA by using qPCR and LAMP in wild long-tailed macaques in study of prevalence of herpes B virus in Thailand, 2018–2024*

Sample no.	Age class	Sex	qPCR of UL29 gene			LAMP of UL27 gene	
			Average Ct	Estimated DNA copy no.		Amplification time, h:m:s	Annealing temperature, °C
WTT47	Juvenile	M	37.5	9		0	87.16
WKN63	Subadult	M	36.3	20		0:24:31	89.97
WKN64	Juvenile	F	27.1	5,565		0:14:16	90.21
WKN73	Subadult	F	27.1	5,672		0:14:31	90.35
BTB14	Subadult	M	29.6	1,199		0:15:46	90.36
CAO38	Subadult	F	38.0	7		0:24:46	90.21
CAO41	Subadult	M	26.7	7,074		0:15:01	90.36
TPK5	Juvenile	M	32.1	1,607		0:15:31	90.36
MFRC17	Adult	M	25.7	12,579		0:13:16	90.01

*Ct, cycle threshold; h:m:s, hours:minutes:seconds; LAMP, loop-mediated isothermal amplification; qPCR, quantitative PCR.

Nine monkeys from 6 populations had positive qPCR results. When we analyzed B virus shedding according to subspecies, the virus detection rate for *M. fascicularis* subsp. *aurea* (2.17% [2/92]) was slightly, but not significantly, higher than that for *M. fascicularis* subsp. *fascicularis* (0.90% [7/772]) (Table 3). The averaged cycle thresholds ranged from 25.7 to 38.0, and estimated DNA copy numbers ranged from 7 to 12,579 by qPCR for those 9 monkeys (Table 5). The *M. fascicularis* subsp. *fascicularis* macaque population from Wat KuPhra Ko Na (WKN) had the highest B virus shedding rate of 3.89% (3/77) by both detection methods. An *M. fascicularis* subsp. *aurea* macaque from the Mangrove Forest Research Center (no. MFRC17) had the highest estimated DNA copy number, 12,579. Although the subadult monkeys appeared to have a higher B virus shedding rate (1.76% [5/284]), correlation analysis revealed no significant differences in shedding among the 3 age classes or sexes (Table 6), likely because the number of qPCR-positive cases was low.

**Table 6:** Detection of B virus shedding in wild long-tailed macaques by using qPCR in study of prevalence of herpes B virus in Thailand, 2018–2024*

Age class	No. positive/total (%, 95% CI)			
	Male	Female	Total	
Adult	1/240 (0.42, 0–1.7)	0	1/393 (0.26, 0–0.9)	
Subadult	3/177 (1.69, 0–7.7)	2/107 (1.87, 0–2.6)	5/284 (1.76, 0–4.5)	
Juvenile	2/111 (1.80, 0–4.1)	1/76 (1.32, 0–1.6)	3/187 (1.60, 0–2.4)	
Total	6/528 (1.14, 0–3.5)	3/336 (0.89, 0–1.1)	9/864 (1.04, 0.2–2.0)	

*qPCR, quantitative PCR.

Of the 9 qPCR-positive samples, we successfully amplified only 6 samples (nos. WTT47, WKN63, WKN64, WKN73, CAO41, and TPK5) by using nested PCR with UL29 and UL27 primers. The B virus sequences from those 6 samples were 99.35%–100% homologous to the B virus reference sequence, E90-136 (data not shown).

### Phylogenetic Analysis of US5-US6 B Virus Sequences

**Figure 1:**
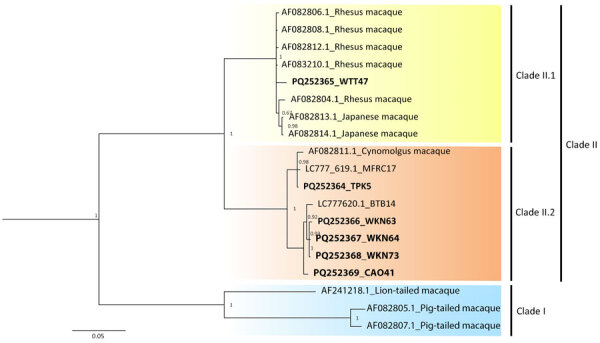
Phylogenetic analysis of herpes B virus in wild long-tailed macaques, Thailand, 2018–2024. Maximum-likelihood trees of partial B virus genome sequences (US5-US6 genes) were constructed for 8 long-tailed macaques from 6 populations in Thailand. Bold font indicates 6 sequences from this study compared with 2 sequences previously described (LC777620.1_BTB14 and LC777619.1_MFRC17) (*14*). Values on each branch indicate the posterior probability/bootstrap value. Scale bar indicates nucleotide substitutions per site.

**Figure 2:**
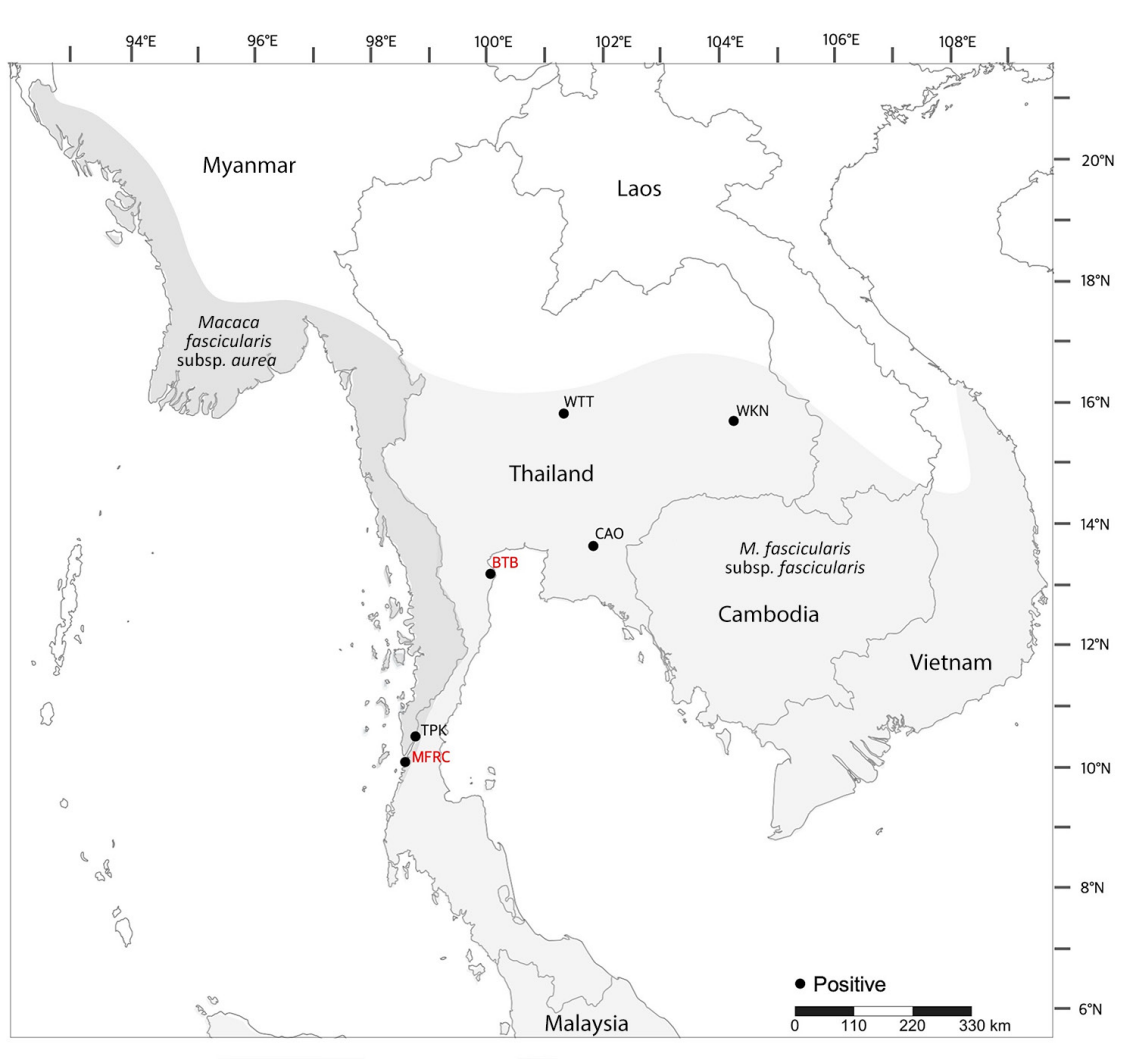
Distribution of 6 virus-positive wild long-tailed macaque populations in study of prevalence of herpes B virus in Thailand, 2018–2024. Dark gray shading indicates distribution of *Macaca fascicularis* subspecies *aurea* macaques; light gray shading indicates geographic range of *M. fascicularis* subsp. *fascicularis* macaques. Red text indicates locations of macaques BTB14 and MFRC17 described previously (*14*). BTB, Bang Ta Boon; CAO, Wat Tham Khao Cha Ang On; MFRC, Mangrove Forest Research Center; TPK, Tham Pra Khayang; WKN, Wat KuPhra Ko Na; WTT, Wat Tham Thep Ban Dan.

We successfully amplified and sequenced 6 of 9 qPCR-positive oral swab samples by using the US5-US6 primer set (734-bp amplicon). We submitted the sequences to GenBank (accession nos. PQ252364–9). The phylogenetic tree revealed 2 major clades: clade I, lion-tailed/southern pig-tailed macaques; and clade II, rhesus/Japanese/long-tailed macaques (Figure 1). Clade II was divided further into clade II.1 (rhesus/Japanese macaques) and clade II.2 (long-tailed macaques). All B viruses detected in this study were clustered in clade II; 1 B virus sample (detected in a *M. fascicularis* subsp. *fascicularis* macaque from WTT) belonged to clade II.1, and 5 samples (3 *M. fascicularis* subsp. *fascicularis* macaques from WKN and 1 from Wat Tham Khao Cha Ang On and 1 *M. fascicularis* subsp. *aurea* macaque from Tham Pra Khayang) belonged to clade II.2 (Figures 1, 2). Clade II.2 also included the previously reported B viruses detected in *M. fascicularis* subsp. *fascicularis* (LC777620.1_BTB14) and *M. fascicularis* subsp. *aurea* (LC777619.1_MFRC17) macaques (Figures 1, 2) (*14*). Within a single population from WKN, all 3 B virus sequences (nos. WKN64, WKN73, and WKN63) had 100% homology.

## Discussion

*M. mulatta* and *M. fascicularis* macaques are commonly used as nonhuman primate models for testing biopharmaceutical drugs. All confirmed human cases of zoonotic B virus have been in persons working with those captive macaque species (*10*), which were imported from Asia or were the offspring of macaques originally exported from Asia. Cases of zoonotic B virus infection in humans caused by contact with wild macaques from Asia have not been reported in the scientific literature. It remains unclear why highly pathogenic B virus does not appear to be present in or transmitted to humans who have contact with wild macaques in their natural habitats (*10*).

We provide critical information regarding the spread of B virus in 22 macaque populations across Thailand, which has implications for both animal health and zoonotic disease risk (*26*). We used ELISAs for antibody detection and qPCR and LAMP to identify virus DNA. The advantage of ELISA is its speed and accuracy in assessing exposure and immune response to the virus, making it useful for large-scale epidemiologic studies. However, this serologic test cannot measure active virus shedding or potential transmission risk because it only detects antibodies that can remain in the host’s system long after the initial infection has resolved (*27*). That limitation can be addressed by using qPCR and LAMP. Both qPCR and LAMP consistently identified B virus–positive samples, indicating their reliability in detecting active viral shedding. We previously reported that the detection limit was 50 DNA copies/reaction for LAMP and 10 DNA copies/reaction for qPCR (of UL29) (*14*). Our findings confirmed that qPCR using UL29 primers exhibited high sensitivity and could detect as few as 7 virus DNA copies (monkey no. CAO38). Despite LAMP having lower sensitivity than qPCR, its rapid turnaround time and ease of use make it a valuable tool for preliminary screening, especially in resource-limited settings such as field studies.

We found a high prevalence of B virus antibodies among *M. fascicularis* macaques in Thailand; 78.5% of the 864 monkeys sampled were antibody positive. The widespread exposure to B virus within macaque populations has considerable implications for potential virus transmission. The WTT population living on the temple grounds exhibited a 100% positive rate, indicating particularly high transmission intensity in that area. We also found higher B virus antibody prevalence in adults and subadults, suggesting that macaques are lifelong carriers of B virus and older animals have had more exposure to the virus over their lifetimes and during sexual activities (*7*). Our findings are consistent with previous studies demonstrating high B virus prevalence in *M. mulatta* and *M. fascicularis* macaques; 72% prevalence of *M. mulatta* macaques was observed in Puerto Rico (*28*), 64% of *M. mulatta* macaques in Nepal (*29*), and 81.9% of *M. fascicularis* macaques in Indonesia (*30*). In addition, ≈100% of adult *M. mulatta* macaques tested positive for B virus antibodies in Cayo Santiago (*31*,*32*). The B virus seroprevalence did not differ significantly between male (77.5%) and female (80.1%) animals, likely because *M. fascicularis* macaques have a polygynandrous mating system (*15*), and B virus exposure potential should be equal for both sexes.

B virus shedding detected by qPCR was only found in 1.04% of sampled macaques, indicating that active virus shedding was far less common than seropositivity. One possible explanation for this low percentage is that B virus can reactivate and shed from the oral or genital mucosa at a low rate (1%–3%) (*2*,*7*,*10*,*33*), which might be a reason why cases of B virus transmission from wild monkeys to humans in Thailand have not been reported. A 2013–2017 survey of *M. fascicularis* macaque*–*related injuries among locals and tourists from Thailand and foreign countries was conducted in Lopburi Province, where the human-macaque conflict was the greatest in Thailand. A total of 484 persons were injured by macaques, of whom 432 were hospitalized, but no B virus infections were reported (*16*). Nevertheless, the presence of B virus DNA in macaque oral swab samples highlights the potential for transmission through saliva.

Previous studies have suggested that B virus infections in monkeys persist throughout their lives. During the latent phase, the virus remains in the trigeminal ganglia and lumbosacral nerve in 40% of seropositive *M. fascicularis* macaques (*34*) and can be reactivated. Because of human food provisioning and no predators, the number of monkeys in residential areas in countries of Asia has greatly increased (*15*), and the number of persons attacked by wild monkeys has also increased (*16*,*17*), likely elevating the risk for B virus infection. In March 2024, a person injured by wild monkeys at a country park in Hong Kong was infected with B virus and exhibited severe symptoms (*35*,*36*). Taken together, our findings provide insights into the risk for human B virus infection from wild macaques.

In conclusion, the 6 B virus sequences from this study and 2 sequences (no. MFRC17 [*M. fascicularis* subsp. *aurea*] and no. BTB14 [*M. fascicularis* subsp. *fascicularis*]) described previously (*14*) were all clustered within clade II (rhesus/Japanese/long-tailed macaques) in the phylogenetic tree. Of the 8 B virus sequences, only WTT47 was clustered in clade II.1 (rhesus/Japanese macaques). The WTT macaques were morphologically identified as *M. fascicularis* subsp. *fascicularis* and lived at global positioning system coordinates 15°44′N, 101°02′E (Table 1), which was within the proposed hybrid zone between *M. mulatta* and *M. fascicularis* subsp. *fascicularis* macaques (latitude 15°N–20°N) (*22*,*23*,*37*–*39*). Thus, macaques from WTT likely carried genetic material from *M. mulatta* macaques (*23*). Within clade II.2 (long-tailed macaques), *M. fascicularis* subsp. *aurea* macaques from Tham Pra Khayang and Mangrove Forest Research Center grouped separately from *M. fascicularis* subsp. *fascicularis* macaques from Wat Tham Khao Cha Ang On, Bang Ta Boon, and WKN. Our findings support our previous hypothesis that B virus and macaques have co-evolved. B virus had specific infection and incubation mechanisms in the macaque hosts, and each B virus clade has evolved independently in particular macaque populations, species, and subspecies. Continued surveillance and genetic analyses of B viruses in macaque populations will be needed to prevent B virus transmission to humans and develop vaccines to prevent human B virus infections.

Appendix Additional information for prevalence of herpes B virus in wild long-tailed macaques, Thailand, 2018–2024.
