# Supplementary material for: Prevalence of Herpes B Virus in Wild Long-Tailed Macaques, Thailand, 2018–2024
Source: Emerg Infect Dis. 2025 Apr;31(4):741–50. doi: 10.3201/eid3104.241197 (PMC11950252; doi:10.3201/eid3104.241197)
Supplement: Appendix — Additional information for prevalence of herpes B virus in wild long-tailed macaques, Thailand, 2018–2024. [file 24-1197-Techapp-s1.pdf]

Article DOI: https://doi.org/10.3201/eid3104.241197
EID cannot ensure accessibility for supplementary materials supplied by authors. Readers who have difficulty accessing supplementary content should contact the authors for assistance.

Prevalence of Herpes B Virus in Wild Long-Tailed Macaques, Thailand, 2018–2024

Appendix

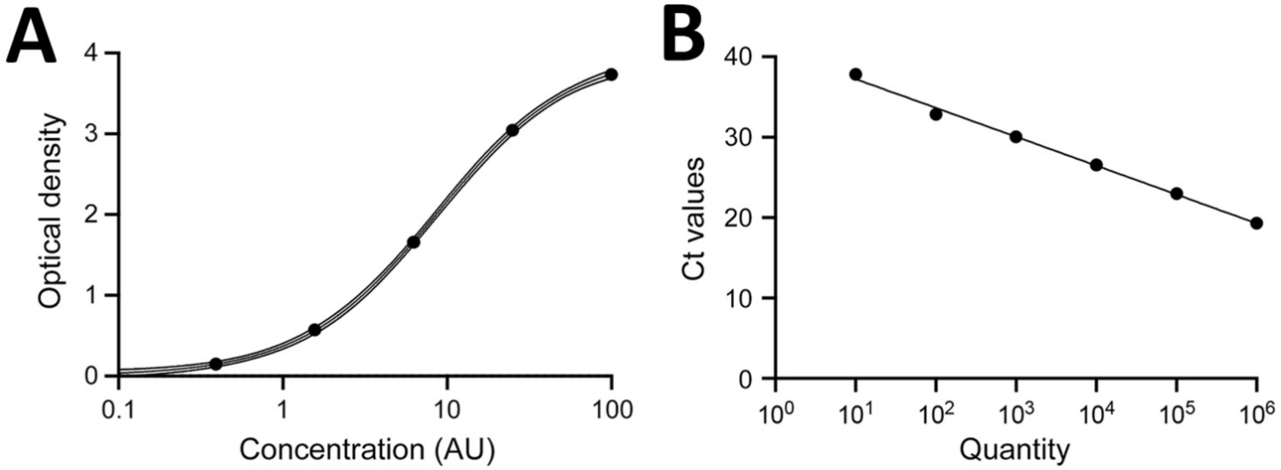

Appendix Figure. Standard curves for ELISA and quantitative PCR used in study of prevalence of herpes B virus in wild long-tailed macaques, Thailand, 2018–2024. A) ELISA standard curve was determined by using a 4-fold dilution series of pooled plasma samples obtained from the National Primate Research Center of Thailand-Chulalongkorn University plasma bank. R^2 = 1.00. B) Quantitative PCR standard curve was determined by using 10-fold serial dilutions of a 257-bp sequence of UL29. Ct cutoff was 40. R^2 = 0.99. AU, arbitrary unit; Ct, cycle threshold.
